# Tracking Insecticide Resistance in Mosquito Vectors of Arboviruses: The Worldwide Insecticide resistance Network (WIN)

**DOI:** 10.1371/journal.pntd.0005054

**Published:** 2016-12-01

**Authors:** Vincent Corbel, Nicole L. Achee, Fabrice Chandre, Mamadou B. Coulibaly, Isabelle Dusfour, Dina M. Fonseca, John Grieco, Waraporn Juntarajumnong, Audrey Lenhart, Ademir J. Martins, Catherine Moyes, Lee Ching Ng, João Pinto, Kamaraju Raghavendra, Hassan Vatandoost, John Vontas, David Weetman, Florence Fouque, Raman Velayudhan, Jean-Philippe David

**Affiliations:** 1 Institut de Recherche pour le Développement (IRD), Maladies Infectieuses et Vecteurs, Ecologie, Génétique, Evolution et Contrôle (MIVEGEC, UM1-CNRS 5290-IRD 224), Montpellier, France; 2 University of Notre Dame (UND), Eck Institute for Global Health, Department of Biological Sciences, Notre Dame, Indiana, United States of America; 3 Malaria Research and Training Center (MRTC), Bamako, Mali; 4 Institut Pasteur de la Guyane (IPG), Cayenne, French Guiana; 5 Center for Vector Biology, Rutgers University (RU), New Brunswick, New Jersey, United States of America; 6 Department of Entomology, Kasetsart University (KU), Bangkok, Thailand; 7 Center for Global Health, Division of Parasitic Diseases and Malaria/Entomology Branch, Centers for Disease Control and Prevention (CDC), Atlanta, Georgia, United States of America; 8 Instituto Oswaldo Cruz (Fiocruz), Rio de Janeiro, Brazil; 9 Oxford Big Data Institute, Li Ka Shing Centre for Health Information and Discovery, University of Oxford (OU), Oxford, United Kingdom; 10 Environmental Health Institute (EHI), National Environment Agency (NEA), Singapore; 11 Global Health and Tropical Medicine, (GHTM), Instituto de Higiene e Medicina Tropical (IHMT), Universidade Nova de Lisboa, Lisboa, Portugal; 12 Insecticides and Insecticide Resistance Lab, National Institute of Malaria Research (NIMR), Delhi, India; 13 Department of Medical Entomology & Vector Control, School of Public Health, Tehran University of Medical Sciences (TUMS), Tehran, Iran; 14 Institute Molecular Biology and Biotechnology (IMBB), Foundation for Research and Technology (FORTH), Crete, Greece; 15 Pesticide Science Lab, Agricultural University of Athens, Athens, Greece; 16 Vector Biology Department, Liverpool School of Tropical Medicine (LSTM), Liverpool, United Kingdom; 17 The Special Programme for Research and Training in Tropical Diseases, World Health Organization, Geneva, Switzerland; 18 Vector Ecology and Management, Department of Control of Neglected Tropical Diseases (HTM/NTD), World Health Organization, Geneva, Switzerland; 19 Centre National de la Recherche Scientifique (CNRS), Laboratoire d'Ecologie Alpine (LECA), UMR 5553, Université de Grenoble, Domaine universitaire de Saint Martin d'Hères, Grenoble, France; Centers for Disease Control and Prevention, Puerto Rico, UNITED STATES

## Context: Current Strategies and Challenges for Arbovirus Control

The transmission of the arboviral agents of dengue, yellow fever, Chikungunya, and Zika by *Aedes* mosquitoes represents expanding threats to global health. At the 69th World Health Assembly [1], the WHO Director-General Margaret Chan declared that the spread of the Zika virus was "the result of the abandon of mosquito control" by governments since the 1970s and urged Member States to mobilize more efforts and resources to prevent further spread of the diseases. The recent rise of microcephaly cases and other neurological disorders reported in Brazil prompted WHO to declare Zika as a Public Health Emergency of International Concern [2]. After limited early outbreaks in the Pacific in 2007 and 2013, the Zika virus has spread to more than 30 countries in the Americas and the Caribbean, affecting over 1.5 million people [3]. With growing evidence supporting the link between microcephaly and Zika [4, 5] and preliminary evidence confirming *Aedes aegypti* as the primary vector in the Brazilian outbreak [6], the mandate for control is clear and urgent.

Although progress is being made on vaccine development (for example, Sanofi Pasteur’s recently licensed dengue vaccine Dengvaxia [7]), vector control by removing larval habitats and using biological and chemical insecticides still remain the first line of defence against arboviruses [8]. Unfortunately, decades of efforts failed to consistently control *Aedes* mosquito populations and/or to curtail the cycle of epidemics. Control of adult mosquitoes using space spray applications of pyrethroids and organophospates in plural is fraught with complications, including high cost, slow operational response, low community buy-in, ineffective timing of application, and rather low efficacy and/or residual effect [9–11]. Furthermore, some countries have a lack of capacity in monitoring the use of public health insecticides for the control of arbovirus vectors [12] that is essential for guiding pesticide management systems on appropriate use and reduction of risks to human health and environment.

In spite of the growing international concern, control of *Aedes*-borne arboviral diseases is hindered by financial constraints. An estimated US$9,900,000,000 has been committed by international donor agencies for malaria control in endemic countries between 2002 and 2010 [13]. Conversely, vector control interventions targeting arbovirus vectors remain under the financial and logistical responsibility of national programmes, which are funded from national budgets with no sustained external funding sources. Research on the discovery of novel insecticides as well as new paradigms for mosquito control is ongoing [14], but organized vector control still relies primarily on just two chemical classes of insecticides (namely pyrethroids and organophosphates). This is largely due to the perceived limitations in the public health market and lack of industry incentives. The use of the same insecticides for more than 40 years coupled with the extensive traffic of *Aedes* eggs has resulted in the worldwide spread of insecticide resistance [15]. Resistance is now considered by WHO as a major threat for the control of diseases transmitted by mosquitoes and has likely contributed to the reemergence and/or spread of arboviruses.

## The WIN Initiative: A Global Approach to Combat Insecticide Resistance in Arbovirus Vectors

A coordinated approach is imperative to detect and manage insecticide resistance at the early stage and to deploy alternative strategies for vector control. Institutions and stakeholders have to collaborate in an integrated manner to improve the research and training capacity of national partners located in endemic areas and countries faced with outbreak. Supported by the WHO Special Programme for Research and Training in Tropical Diseases (TDR) and the Department of Neglected Tropical Diseases (NTDs) since March 2016, the Worldwide Insecticide resistance Network (WIN, http://win-network.ird.fr) brings together 16 internationally recognized institutions in vector research from Africa, the Eastern Mediterranean, Europe, South America, Southeast Asia, North America, and the Western Pacific to track insecticide resistance at a global scale. The overall goal of WIN is to provide WHO and Member States with evidence and expertise to support recommendations for resistance management and deployment of alternative arbovirus vector control methods. Specific objectives are to identify regions and countries where insecticide resistance may challenge mosquito control, to explore the mechanisms conferring resistance, and to predict further expansion. Such objectives will be achieved by producing in-depth reviews of insecticide resistance–related topics by internationally recognized experts.

## WIN Expectations: Capacity Building and Strengthening for Monitoring of Insecticide Resistance in Arbovirus Mosquito Vectors

The WIN network will facilitate the engagement of scientists, stakeholders, members of the private and public sectors, and decision makers from around the world to share knowledge and ideas. An international workshop is planned in Rio de Janeiro, Brazil, on December 5–8th, 2016, to raise awareness and mobilize resources for strengthening the capacity of national authorities in arbovirus vector control and to facilitate basic and translational research with the scope to improve vector control and management of insecticide resistance (http://win-network.ird.fr). The objectives of the workshop will be to (i) share knowledge and information on distribution, mechanisms, and impact of insecticide resistance in invasive mosquito vectors; (ii) review the new tools and strategies for the control of insecticide-resistant arbovirus vectors; (iii) promote private–public partnership for the development of new insecticide products; and (IV) guide national authorities in the development of strategic plans for vector control and sound management of pesticide use in public health. At the time of writing this article, ten countries have provided financial assistance or have pledged support to the WIN network, but long-term development and sustainability of this initiative will require further financial support.
